# Evaluating *Streptococcus mutans* Strain Dependent Characteristics in a Polymicrobial Biofilm Community

**DOI:** 10.3389/fmicb.2018.01498

**Published:** 2018-07-23

**Authors:** Yan Zhou, Emma Millhouse, Tracy Shaw, David F. Lappin, Ranjith Rajendran, Jeremy Bagg, Huancai Lin, Gordon Ramage

**Affiliations:** ^1^Oral Sciences Research Group, Glasgow Dental School, School of Medicine, Dentistry and Nursing, College of Medical, Veterinary and Life Sciences, University of Glasgow, Glasgow, United Kingdom; ^2^Department of Preventive Dentistry, Guanghua School of Stomatology, Sun Yat-sen University, Guangzhou, China; ^3^ESCMID Study Group for Biofilms, Basel, Switzerland

**Keywords:** *Streptococcus mutans*, biofilm, polymicrobial, caries, models

## Abstract

**Aim:** The purpose of this study was to investigate strain dependent differences of the cariogenic biofilm forming *Streptococcus mutans* within both simple and complex communities.

**Methods:** A mono-species containing representative *S. mutans* clinical isolates (caries and non-caries), and a multispecies *in vitro* caries biofilm model containing *Lactobacillus casei*, *Veillonella dispar*, *Fusobacterium nucleatum* and *Actinomyces naeslundii*, and either of two representative *S. mutans* clinical isolates (caries and non-caries), was developed as a comparison model. Compositional analysis of total and live bacteria within biofilms, and transcriptional analysis of biofilm associated virulence factors were evaluated by live/dead PCR and quantitative PCR, respectively. Scanning electron microscopy (SEM) was used to analyze the architecture of biofilm. One-way analysis of variance and *t*-tests were used to investigate significant differences between independent groups of data.

**Results:** Within a mono-species biofilm, different *S. mutans* strains responded similarly to one another during biofilm formation in different carbohydrate sources, with sucrose showing the highest levels of biofilm biomass and galactose showing the lowest. Within the polymicrobial biofilm system, compositional analysis of the bacteria within the biofilm showed that *S. mutans* derived from a caries-free patient was preferentially composed of both total and viable *L. casei*, whereas *S. mutans* derived from a caries patient was dominated by both total and viable *S. mutans* (*p* < 0.001). Normalized gene expression analysis of *srtA*, *gtfB*, *ftf*, *spaP*, *gbpB*, and *luxS*, showed a general upregulation within the *S. mutans* dominant biofilm.

**Conclusion:** We were able to demonstrate that individual strains derived from different patients exhibited altered biofilm characteristics, which were not obvious within a simple mono-species biofilm model. Influencing the environmental conditions changed the composition and functionality *S. mutans* within the polymicrobial biofilm. The biofilm model described herein provides a novel and reproducible method of assessing the impact on the biofilm microbiome upon different environmental influences.

## Introduction

*Streptococcus mutans* has been reported as a primary cariogenic bacterial pathogen (Loesche, 1986). A key virulence attribute of *S. mutans* exists in its capacity to metabolize and process various sugar carbohydrates, and use these to form biofilms on the enamel surface of the tooth (Bowen and Koo, 2011). These biofilms are largely composed of bacterial microcolonies embedded in an extracellular polymeric matrix. The matrix prevents against mechanical removal and aids in defense against host protein interactions, additionally acting as a diffusion barrier from a range of natural and artificial antimicrobial agents (Senadheera and Cvitkovitch, 2008). The process of carbohydrate metabolism and development of the biofilm facilitates a disadvantage to the host. Environmental pH is driven down within the oral cavity leading to demineralization of hydroxyapatite, enamel dissolution, and ultimately carious lesions (Takahashi and Nyvad, 2008). However, this is a rather over simplistic viewpoint, despite the well-established multifactorial basis of this disease. Several studies have revealed that the level of *S. mutans* is not necessarily high in caries-associated biofilms, suggesting that the microbial basis of this disease is subtler, and yet more complicated at the same time. Indeed, the increasing acid environment facilitates increased proportions of different *S. mutans* genotypes and other acidogenic species (Napimoga et al., 2005). The interconnectedness of *S. mutans* biofilms and overall ecology within the oral environment is likely to play a critical role in driving disease phenotypes. Recent evidence suggests the presence of specific functional ecotypes with either a saccharolytic or proteolytic propensity may be the driving factors in oral biofilm diseases (Zaura et al., 2017).

*S. mutans* is a diverse species of bacteria, and this variety has the potential to influence adhesion, biofilm formation and associated virulence (Napimoga et al., 2005). Indeed, isolates of *S. mutans* are known to vary in a range of phenotypic properties (Lembo et al., 2007). Palmer and colleagues sequenced 57 geographically and genetically diverse isolates of *S. mutans*, where a high degree of variability in properties were observed between strains, including a broad spectrum of sensitivities to low pH, oxidative stress, and exposure to competence stimulating peptide (Palmer et al., 2013). Moreover, our previous study highlights the genotypic diversity of *S. mutans* isolates from children and indicates that *S. mutans* heterogeneity may correlate with caries susceptibility (Yu et al., 2015). These genotypic variations in adhesion, colonization and associated pathogenicity are thought to underlie the known individual variation in cariogenicity, though until now, attempts to correlate them with specific individual properties with caries in humans have been largely unsuccessful. Therefore, a significant research effort toward understanding the mechanisms of the development of mixed-species communities containing *S. mutans*, and defining their active role, are required. The advent of microbiome analysis has started to reveal the relative importance of different bacterial species within the community structure, though meta-transcriptomic approaches have been more informative in showing active participation. Indeed, recent reports suggest that *S. mutans* actively represented only 0.02–0.73% of the bacterial community in disease, exemplifying the polymicrobial nature of this disease (Simon-Soro et al., 2014). This further supports the ecological plaque hypothesis of dental caries, where the entire consortium of acidogenic/aciduric bacteria, not only the mutans streptococci, contributes to the caries process (Takahashi and Nyvad, 2011). The recent characterization of saccharolytic ecotypes in otherwise orally healthy individuals suggests our own functional microbiome plays a role in dictating whether or not we are caries susceptible (Zaura et al., 2017). To facilitate further understanding of these mechanisms, models capable of dissecting and elucidating biological processes with caries are necessary. Current models have limited biodiversity with a lack of representation of other species and genus; therefore are not representative of competing bacteria that may be present within a cariogenic state. To this end, the purpose of this study was to develop a suitable caries biofilm model that was able to recapitulate these aforementioned concepts, i.e., the biological diversity associated with *S. mutans* clinical isolates, and test how this translates within a simple mono-species and novel polymicrobial biofilm system.

## Materials and Methods

### Ethics Statement

Isolates of *S. mutans* used in this study were obtained from a protocol approved by the Ethics Committee of Guanghua School of Stomatology, Sun Yat-sen University (ERC-[2012]-13).

### Assessing Mono-Species Biofilm Development of Clinical Isolates

A subset of *S. mutans* clinical strains isolated from children without caries were used for this study (Yu et al., 2015). *S. mutans* (*n* = 20) were selected to form the caries-free group, and *S. mutans* from children with a DMFT index of ≥6 (*n* = 105) were selected to form the caries group. All *S. mutans* clinical isolates were propagated on horse blood agar (E & O Laboratories Ltd., Bonnybridge, United Kingdom) at 37°C in 5% CO_2_ for purity plating, then single colonies incubated in brain heart infusion (BHI) broth overnight at 37°C in 5% CO_2_ prior to standardization to 1 × 10^7^ cells/mL. *S. mutans* strains from the caries (*n* = 10) and caries-free (*n* = 10) isolates were then assessed for biofilm growth in different carbohydrate sources [sucrose, mannose, fructose, glucose and galactose (Sigma-Aldrich, Dorset, United Kingdom)] at 0.125, 0.5, 1, 2, and 5% (w/v) in BHI. Mono-species biofilms were grown in flat-bottomed 96-well microtiter plates at 37°C in 5% CO_2_ for 24 h. Following incubation, the supernatant was removed for pH testing using a pH microelectrode (Mettler Toledo, Switzerland), and the resultant biofilm biomass was quantified using a crystal violet (CV) stain, as described previously (Millhouse et al., 2014; Sherry et al., 2016). Briefly, biofilms were dried overnight at room temperature before staining with 0.05% (w/v) CV (Sigma-Aldrich, Dorset, United Kingdom) for 15 min. The stained biofilms were then washed with water to remove excess dye, then de-stained with 100% ethanol. The released dye was then quantified in a plate reader (FluoStar Omega, BMG Labtech) at 570 nm. To measure biofilm viability, AlamarBlue^®^ (Invitrogen, Paisley, United Kingdom) was added to PBS washed biofilms and incubated for 1 h in 1:10 AlamarBlue^®^:BHI media at 37°C in 5% CO_2_, prior to a color change being quantified on a plate reader at 570/600 nm, according to manufacturer’s instructions. Each biofilm was evaluated three times, and the mean recorded as pH values. All analysis was performed in triplicate on at least three separate occasions. Scanning electron microscopy (SEM) was performed on selected biofilms to visualize the effects of selected carbohydrates. Following biofilm development, cells were carefully washed with PBS, fixed in 2% paraformaldehyde, 2% glutaraldehyde, 0.15M sodium cacodylate, and 0.15% w/v Alcian Blue (pH 7.4), as previously described (Erlandsen et al., 2004). The specimens were sputter-coated with gold and viewed under a JEOL JSM-6400 scanning electron microscope. Images were assembled using Photoshop software (Adobe, San Jose, CA, United States).

### Development of a Multi-Species Caries Biofilm Model

For multi-species biofilm model studies, a selection of laboratory strains commonly associated cariogenic bacterial species were used. These included *Fusobacterium nucleatum* ATCC 10596, *Veillonella dispar* ATCC 27335, *Actinomyces naeslundii* ATCC 19039 and *Lactobacillus casei* DSMZ 20011. All procedures were carried out to BSL-2 safety requirements. Two *S. mutans* clinical isolates were also included (caries and non-caries) in comparison models. *S. mutans* and *L. casei* were maintained on Colombia blood agar [CBA; Oxoid, Hampshire, United Kingdom] and MRS Agar (Sigma-Aldrich, Dorset, United Kingdom) at 37°C in 5% CO_2_, respectively. *V. dispar*, *A. naeslundii and F. nucleatum* were all maintained on fastidious anaerobic agar [FAA; Lab M Ltd., Lancashire, United Kingdom] under anaerobic conditions (85% N_2_, 10% CO_2,_ and 5% H_2_, [Don Whitley Scientific Limited, Shipley, United Kingdom]). Overnight cultures of *S. mutans* and *L. casei* were grown for 24 h in BHI broth and MRS broth at 37°C in 5% CO_2_, respectively. Cultures of *A. naeslundii* and *V. dispar* were grown in BHI and *F. nucleatum* was cultured in Schaedler’s anaerobic broth [Oxoid, Basingstoke, United Kingdom], and all were incubated for 48 h anaerobically (85% N_2_, 10% CO_2_, and 5% H_2_). All bacterial cultures were standardized to a final concentration of 1 × 10^8^ cells/mL by washing, centrifuging and resuspending in PBS.

A multi-species caries biofilm model was created by adopting a similar approach to our previously published studies (Millhouse et al., 2014; Sherry et al., 2016). Briefly, standardized *S. mutans*, *L. casei*, *V. dispar*, *F. nucleatum*, and *A. naeslundii* were diluted 1:10 to 1 × 10^7^ cells/mL in artificial saliva (AS) containing either sucrose and galactose (Sigma-Aldrich, Dorset, United Kingdom) at a single defined concentration of 1% w/v, and added to a 24-well tissue culture plate (Corning, NY, United States) containing 13 mm^2^ × 1.5 mm thick hydroxyapatite coverslips (Plasma Biotal Ltd., Tideswell, Derbyshire, United Kingdom). The bacterial suspensions were then incubated statically at 37°C in 5% CO_2_ for 120 h, with spent supernatants being replaced with fresh AS every 24 h. Biofilms were then assessed for both biomass and viability, using CV and AlamarBlue^®^ assays, respectively, as described above.

### Compositional Analysis of Total and Live Bacteria Within Biofilms

To enumerate the viable and total number of cells from the biofilm a qPCR viability method was used, as previously described by our group (Sherry et al., 2016). Briefly, multi-species biofilms were prepared as described above, from 24 to 120 h, in sucrose and galactose. Resultant biofilms were gently washed in PBS prior to sonication from the HA surface at 35 kHz for 10 min. The sample was split into two equal homogenous suspensions and to one of these 50 μM of propidium monoazide (Sigma-Aldrich, Dorset, United Kingdom) was added prior to incubation for 10 min in the dark (viable cells). This was then exposed for 5 min to a 650 W halogen light. The remaining sample without the addition of propidium monoazide was used as controls to quantify the biomass (total cells). To extract the DNA the QIAmp mini DNA Extraction Kit (Qiagen, Crawley, United Kingdom) was used, following the manufacturer’s instructions with the modification that the sonicate was incubated for 2.5 h to ensure cell lysis and the addition of mutanolysin (Sigma-Aldrich, Dorset, United Kingdom). Real-time quantitative (qPCR) was then performed to quantify the composition of biofilms in both the total and live only samples following treatment. For qPCR, 1 μL of extracted DNA was added to a mastermix containing 10 μL SYBR^®^ GreenER^TM^, 7 μL UV-treated RNase-free water and 1 μL of 10 μM forward/reverse primers for each bacterial species. The primers used were previously published and are listed in **Table 1**. The thermal profile used consisted of an initial denaturation of 95°C for 10 min followed by 40 cycles of 30 s at 95°C, 60 s at 55°C, and 60 s at 72°C. Three independent replicate using MxProP Quantitative PCR machine and MxPro 3000P software (Stratagene, Amsterdam, Netherlands). Samples were quantified to calculate the colony forming equivalent (CFE) based upon a standard curve methodology of bacterial colony forming units ranging from 1 × 10^4^ to 10^8^ CFU/mL. Melting curve analysis was performed for all primer sets to ensure a single peak, which was indicative of primer specificity.

**Table 1:** Sequences of primers used in this study.

Target	Primer Sequence (5’-3’)	Reference
**Bacterial species (16S rRNA)**		
*Streptococcus spp.*	F – GATACATAGCCGACCTGAG	Bernabé et al., 2016


	R – CCATTGCCGAAGATTCC	
*A. naeslundii*	F – GGCTGCGATACCGTGAGG	Periasamy et al., 2009


	R – TCTGCGATTACTAGCGACTCC	
*F. nucleatum*	F – GGATTTATTGGGCGTAAAGC	Bernabé et al., 2016


	R – GGCATTCCTACAAATATCTACGAA	
*V. dispar*	F – CCGTGATGGGATGGAAACTGC	Periasamy et al., 2009


	R – CCTTCGCCACTGGTGTTCTTC	
*L. casei*	F – TGCACTGAGATTCGACTTAA	Desai et al., 2006


	R – CCCACTGCTGCCTCCCGTAGGAGT	
***S. mutans* gene**		
*spaP*	F – TCCGCTTATACAGGTCAAGTTG	Bernabé et al., 2016


	R – GAGAAGCTACTGATAGAAGGGC	
*gbpB*	F – CGTGTTTCGGCTATTCGTGAAG	Bernabé et al., 2016


	R – TGCTGCTTGATTTTCTTGTTGC	
*gtfB*	F – AGCAATGCAGCCAATCTACAAAT	Bernabé et al., 2016


	R – TACGAACTTTGCCGTTATTGTCA	
*luxS*	F – ACTGTTCCCCTTTTGGCTGTC	Bernabé et al., 2016


	R – AACTTGCTTTGATGACTGTGGC	
*srtA*	F – GAAGCTTCCTGTAATTGGCG	Levesque et al., 2005


	R – TTCATCGTTCCAGCACCATA	
*Ftf*	F – AAATATGAAGGCGGCTACAACGC	Bernabé et al., 2016


	R – CTTCACCAGTCTTAGCATCCTGAA	


### Transcriptional Analysis of Biofilm Associated Virulence Factors

Real-time quantitative PCR was used to assess biofilm associated changes during biofilm formation with 1% sucrose. Biofilms were sonicated (Ultrasonic bath, Fisher scientific, United Kingdom) from the surface of the HA at 35 kHz for 10 min to harvest the cells. These were then washed by centrifugation prior to RNA extraction using a combined mechanical disruption (0.5 mm glass beads) and chemical TRIzol^TM^ method (Invitrogen, Paisley, United Kingdom). After DNase treatment (Qiagen, Crawley, United Kingdom) and purification (RNeasy MinElute clean up kit, Qiagen, Crawley, United Kingdom), cDNA was synthesized using a High Capacity RNA to cDNA kit (Life Technologies, Paisley, United Kingdom), and quantitative PCR performed using a SYBR^®^ GreenER^TM^ assay (Life Technologies Ltd., Paisley, United Kingdom). The primers used for quantitative PCR were spaP (surface protein), gbpB (glucan binding protein B), gtfB (glucosyltransferases B), srtA (sortase A), luxS (S-ribosylhomocysteine lyase), ftf (fructosyltransferase) and 16S rRNA (**Table 1**). Each parameter was analyzed in duplicate using MxProP Quantitative PCR machine and MxProP 3000 software (Stratagene, Amsterdam, Netherlands). Gene expression was normalized to the housekeeping gene 16S rRNA according to 2^-ΔΔC_T_^ method and percentage of gene expression is shown as log_10_ mean ± SD (Livak and Schmittgen, 2001). A heat map was generated for the percentage expression of genes (log_2_) over the period of 24 to 120 h from the caries compared to caries-free group. Heat map was generated in R programming environment using the heatmap.2 function from the g-plots package.

### Statistical Analysis

Data distribution, graph production and statistical analysis were performed using GraphPad Prism (version 5; La Jolla, CA, United States). After assessing whether data conformed to a normal distribution, One-way Analysis of Variance (ANOVA) and *t*-tests were used to investigate significant differences between independent groups of data that approximated to a Gaussian distribution. A Bonferroni correction was applied to the *p* value to account for multiple comparisons of the data. Any non-parametric data was analyzed using the Mann–Whitney *U*-test or the Kruskal–Wallis test with a Dunn’s post-test to assess differences between independent sample groups. Statistical significance was achieved if *p* < 0.05. A heatmap was created for the differential expression of genes (log_2_) over the period of 24, 72, and 120 h from the caries and non-caries *S. mutans* containing polymicrobial biofilms. Maps and clusters were generated in R with the use of heatmap.2 function from the g-plots package. All experiments were performed in triplicate on three independent occasions.

## Results

### *Streptococcus mutans* Mono-Species Biofilm Influenced by Carbohydrate Source

**FIGURE 1:**
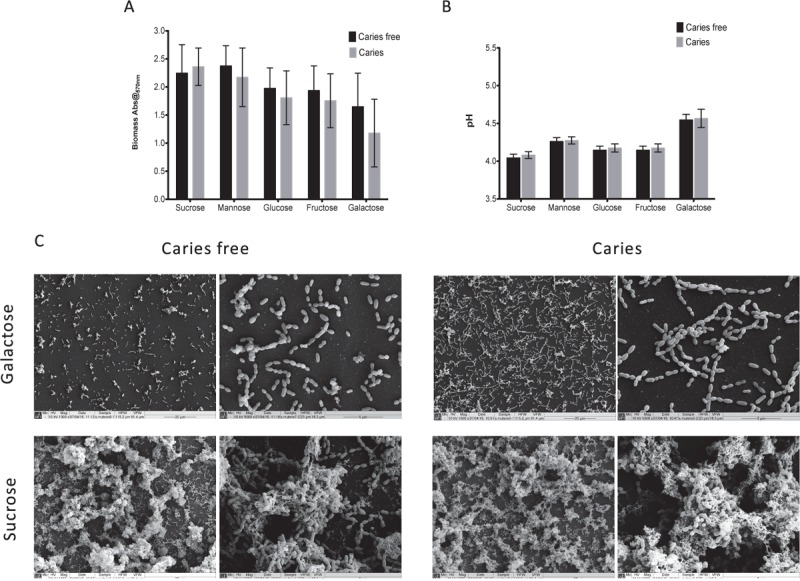
Monospecies cariogenic biofilm biomass is influenced by carbohydrate source. Biomass was quantified spectrophotometrically by reading absorbance at 570 nm in a microtiter plate reader (FluoStar Omega, BMG Labtech). Four replicates were used for each isolate and was carried out on triplicate separate occasions, with the mean of each represented. Data represents mean with significance ^∗^*p* < 0.05. **(A)** Ten isolates with caries and caries free were standardized to 1 × 10^7^ cells/mL in BHI with different carbohydrate and grown as biofilms in flat-bottomed 96 well microtiter plates for 24 h. Different concentrations of sucrose, mannose, fructose, glucose, galactose were added into BHI media (1.0% w/v). Biomass quantified by crystal violet staining. Biomass from caries group and caries free group were compared. **(B)** Supernatant of the mature biofilm were collected and the pH values of them were detected by pH meter. pH values from caries group and caries free group were compared. **(C)** Biofilms were also analyzed by SEM at both 1000× and 5000×. Samples were processed and viewed on a JEOL JSM-6400 scanning electron microscope and images assembled using Photoshop software. Strains from caries free group were grown in 1% galactose and 1% sucrose were compared with caries group separately.

We first screened a large panel of *S. mutans* isolates derived from a clinical study under a standardized biofilms assay conditions (Yu et al., 2015). From these we randomly selected 10 isolates from caries patients and 10 from non-caries patients that were closest to the arithmetic mean (**Supplementary Figure S1**). The aim of this approach was to minimize any exaggerated biofilm formation effects associated with enhanced or reduced fitness within this heterogenous panel of clinical isolates. First, we aimed to assess and compare the ability of *S. mutans* to form mono-species biofilms in the presence of different dietary carbohydrates to determine whether we could observe strain dependent differences as mono-species biofilms. Initially, a range of concentrations were evaluated (0.125, 0.25, 0.5, 1, 2, and 5% w/v) in terms of biomass and pH, with the resultant biofilm data demonstrating that the carbohydrate source, irrespective of concentration, showed a consistent trend (**Supplementary Figure S2**), i.e., sucrose consistently gave greater biomass and lower pH, whereas galactose displayed the contrasting polarized effects. We therefore focused on the concentration of 1% w/v with caries-free (*n* = 10) and caries (*n* = 10) isolates, a concentration which has been a consistently reported within analogous studies (Zeng et al., 2013; Liu S.S. et al., 2017; Cai et al., 2018). These data showed that although sucrose displayed the greatest biomass and lowest pH, followed by mannose, glucose, fructose and galactose, there were no significant differences between these caries groups in terms of biomass and pH (**Figures 1A,B**). These substrate specific differences are clearly evident when viewed under a SEM (**Figure 1C**), with isolates grown in galactose characterized by a single monolayer of sparse cells and lack of extracellular matrix, whereas isolates grown in sucrose were characterized by a three-dimensional architecture and excessive extracellular matrix and interconnected microcolonies. Overall, mono-species data confirmed that sucrose as a dietary carbohydrate enhanced *S. mutans* mono-species biofilm formation.

### *Streptococcus mutans* Exhibits Differential Activity Within a Polymicrobial Biofilm

**FIGURE 2:**
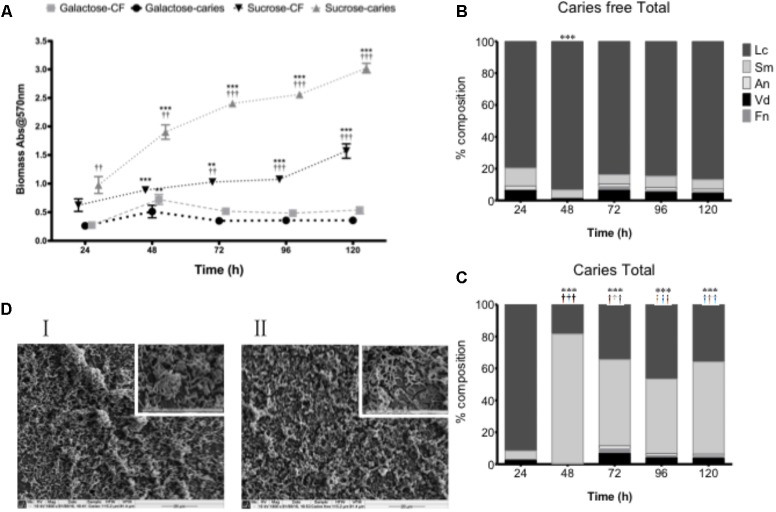
Polymicrobial cariogenic biofilm biomass is influenced by carbohydrate source. **(A)** Polymicrobial caries biofilm model containing standardized *L. casei*, *V. dispar*, *F. nucleatum*, *A. naeslundii* and *S. mutans* isolates derived from either caries and caries-free patients grown at was grown in artificial saliva (AS) + either galactose or sucrose at 37°C in 5% CO_2_ for 5 days, with spent supernatants being replaced with fresh AS every 24 h. Biomass crystal violet staining was quantified spectrophotometrically at 570 nm. Significant increase in growth compared to the 24 h culture (^∗∗^*p* < 0.01, ^∗∗∗^ *p* < 0.001). The total percentage composition of each bacterium grown in AS + 1% sucrose from **(B)** caries free and **(C)** caries derived *S. mutans* was quantified using species-specific qPCR (^∗∗∗^*p* < 0.001). Significant difference between the contribution of *Lactobacillus* at 24 h and other time-points indicated (^∗∗∗^*p* < 0.001). Significant difference between the contribution of *S. mutans* at 24 h and the time-points indicated (^†††^*p* < 0.001). **(D)** Polymicrobial biofilms were analyzed by SEM at ×1,000 and ×5,000. Biofilms from the caries group were dominated by short chains of coccus-shaped bacteria (I) compared to caries free group, which was dominated by rod-shaped bacteria (II).

Although carbohydrates were shown to influence the biofilm architecture and environment as mono-species biofilms, structurally isolates from the caries and caries-free groups could not be phenotypically differentiated. We therefore hypothesized that *S. mutans* biofilm development may be influenced within a more physiologically relevant polymicrobial biofilm environmental state. To test this, we first assessed the total microbial biomass and composition of complex biofilms grown over a 120 h period, where significant growth was observed at each time point compared to 24 h in sucrose (*p* < 0.01) for the caries and non-caries containing biofilms (**Figure 2A**). Biofilms grown in galactose showed no discernable increase in biofilm biomass compared to the 24 h biofilm. Interestingly, significant differences were observed between the caries and caries free isolates grown in sucrose (*p* < 0.01) (**Figure 2A**). Further compositional analysis of the bacteria within the biofilm showed that the caries-free *S. mutans* containing biofilm was preferentially composed of *L. casei* (**Figure 2B**), which dominated throughout the period of growth and development. Whereas, the caries *S. mutans* containing biofilm was initially preferentially composed of *L. casei*, this was subsequently dominated by *S. mutans* from 48 to 120 h (**Figure 2C**). In galactose, *L. casei* dominated each biofilm, irrespective of the *S. mutans* isolate within each biofilm (**Supplementary Figure S3**). SEM analysis of the 120 h polymicrobial biomass from the caries and caries free containing *S. mutans* biofilms showed a dense and complicated intermixed population of morphologically different bacteria. Biofilms from the caries group were dominated by short chains of coccus-shaped bacteria compared to caries free group, which was instead preferentially dominated by rod-shaped bacteria (**Figure 2D**). Both biofilms displayed dense three-dimensional architecture on the hydroxyapatite substrate.

**FIGURE 3:**
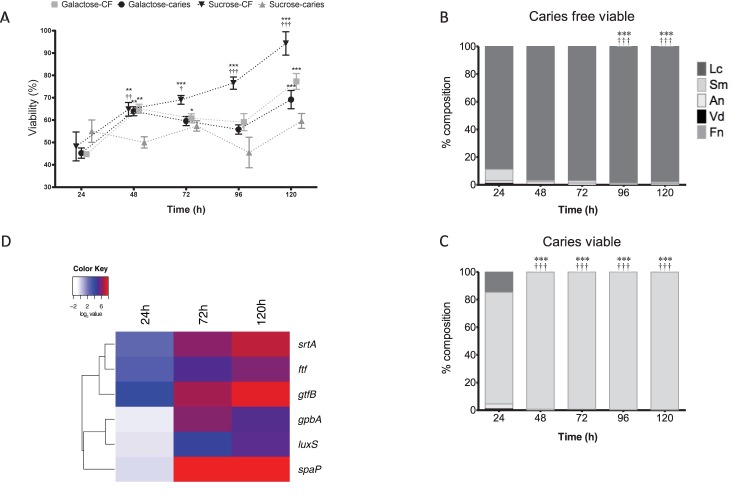
Polymicrobial cariogenic biofilm viability is influenced by carbohydrate source. **(A)** Polymicrobial caries biofilm model containing standardized *L. casei*, *V. dispar*, *F. nucleatum*, *A. naeslundii* and *S. mutans* isolates derived from either caries and caries-free patients grown at was grown in artificial saliva (AS) + galactose or sucrose at 37°C in 5% CO_2_ for 5 days, with spent supernatants being replaced with fresh AS every 24 h. Metabolic activity (AlamarBlue) was quantified spectrophotometrically at 490 nm. Significant increase in growth compared to the 24 h culture (^∗∗^*p* < 0.01, ^∗∗∗^*p* < 0.001,^††^*p* < 0.01). The total viable percentage composition of each bacterium in AS + 1% sucrose from **(B)** caries free and **(C)** caries derived *S. mutans* was quantified using species-specific qPCR (^∗∗∗^*p* < 0.001). Significant difference between the contribution of *Lactobacillus* at 24 h and other time-points indicated (^∗∗∗^*p* < 0.001). Significant difference between the contribution of *S. mutans* at 24 h and the time-points indicated (^†††^*p* < 0.001). **(D)** A heatmap and clustering was created for the differential expression of genes (log_2_). These distinct patterns of gene expression show the related expression profiles of *srtA*, *gtfB*, and *ftf*, and overall upregulation of all genes at 120 h.

Next, we assessed the viability of these polymicrobial biofilms using a metabolic dye and PCR viability assay. A difference is observed in the viability of the caries group and caries free when grown under different sugar conditions. Notably, the *S. mutans* caries isolate containing biofilm showed minimal changes in metabolism when grown in sucrose, whereas the *S. mutans* caries-free isolate containing biofilm showed significantly increased metabolism in a time-dependent manner up to 120 h in comparison to 24 h biofilms (*p* < 0.001), and compared to the other groups tested (*p* < 0.001) (**Figure 3A**). No differences were observed in metabolic activity between the galactose group from caries or caries free, though these from the caries group were significantly increased at 120 h in comparison to the 24 h biofilms (*p* < 0.001) (**Figure 3A**). Furthermore, when these biofilms were investigated compositionally for viable cells, it was noted that *S. mutans* increasingly dominated the biofilms on a daily basis when the isolate was derived from a caries source (**Figure 3B**), whereas the caries-free *S. mutans* polymicrobial biofilm was increasingly dominated daily by viable *L. casei* (*p* < 0.001) (**Figure 3C**). These same analyzes were performed in galactose, however, there is no change between caries and caries free biofilms in terms of the dominating species (**Supplementary Figure S3**).

Given that the biofilms were compositionally distinct depending on the *S. mutans* isolate when grown in 1% sucrose, we then assessed the transcriptional expression of genes deemed important in the adhesion, colonization and associated pathogenicity as shown from the literature (Bernabé et al., 2016). It was shown that in the caries derived *S. mutans* polymicrobial biofilms that *srtA*, *gtfB*, and *ftf* were all upregulated after 24 h (18, 6, and 8%, respectively), 72 h (95, 70, and 32%, respectively) and 120 h (215, 132, and 62%, respectively) compared to non-caries derived biofilms. Whereas, initially at 24 h *spaA*, *luxS*, and *gpbB* were downregulated (0.91, 0.76, and 0.68%, respectively) in the caries derived *S. mutans* polymicrobial biofilms, before being upregulated at 72 h (418, 23, and 65%, respectively) and 120 h (364, 39, and 36%, respectively) compared to non-caries derived biofilms (**Supplementary Figure S4**). These patterns of differential expression are illustrated in the hierarchal clustered log_2_ transformed data, where these distinct patterns of gene expression show the related expression profiles of *srtA*, *gtfB*, and *ftf*, and overall upregulation of all genes at 120 h (**Figure 3D**).

## Discussion

Caries continues to be a significant health problem, particularly amongst those in socially deprived areas, yet is entirely preventable (Pitts et al., 2017). We are all acutely aware that it is multifactorial dynamic disease and is mediated by biofilms. These biofilms are fuelled by sugars, resulting in the periodical phases of demineralization and remineralization of dental hard tissues. Microbiologically, *S. mutans* is recognized as the principal protagonist in caries progression, though its active role has been questioned through some functional transcriptomic techniques. It has been shown that prevalence and composition do not necessarily translate with Koch’s postulates. The data described herein demonstrates that caries microbiology is complex. Mixed consortia of acidogenic bacteria co-exist in biofilms with strain diversity playing an important role in driving the biofilm community structure, particularly under changing sugar conditions. This paper describes a caries polymicrobial biofilm model system that enables careful analysis of the composition and function.

We developed a novel dental caries biofilm model to form either *S. mutans* mono-species biofilms or polymicrobial biofilms including *S. mutans* and a range of bacteria grown in AS. This model is a closed batch culture system. Unlike rotary or continuous systems batch models do offer means of comparing multiple test compounds or conditions simultaneously; they only require small amounts of reagents and are convenient, reproducible, and economical to use (Coenye and Nelis, 2010). One of the most commonly used batch biofilm models is the Zurich biofilm model which uses six microbial species (*Streptococcus oralis*, *S. sobrinus*, *A. naeslundii*, *V. dispar*, *F. nucleatum*, and *Candida albicans*) (Guggenheim et al., 2004). A modification of this model is the three-species version (*S. mutans*, *S. sanguinis*, and *A. naeslundii)* to study the effect of xylitol in a young biofilm (Marttinen et al., 2012). Our model aims to refocus the Zurich biofilm model toward a cariogenic model with the addition of *L. casei*, which is a known antagonist to *S. mutans* (Ahmed et al., 2014). Other cariogenic models often have limited biodiversity and fail to encapsulate the impact of the biofilm in cariogenic outcomes (Chu et al., 2012; Mei et al., 2013; Yu et al., 2017). These models do not allow for the assessment of change in respect to the natural microbiome of the oral cavity. Within the modified model in this study, all the selected bacteria can be found in the process of dental caries (Simon-Soro et al., 2014). The main advantage of this model is its ease of use and requirements for relatively simple equipment, thus providing a robust model for use in the future.

Next, the response of mono-species biofilms to different carbohydrate sources was tested. Dietary carbohydrates were shown to have an impact on the dental caries biofilm model. The disaccharide sugar sucrose showed more cariogenic traits in the process of formation of *S. mutans* biofilm than the comparison monosaccharide sugars. Sucrose stimulated the *S. mutans* biofilm, forming the greatest biomass among the five carbohydrates through enhanced secretion of extracellular polysaccharide (EPS), as confirmed by SEM images. Koo et al. (2010) investigated the relationship between *S. mutans* and exopolysaccharides using the three-species model (*S. mutans*, *S. oralis*, *and A. naeslundii).* In this model ecological changes can be studied with respect to cariogenic biofilm formation. The addition of sucrose resulted in a shift in the proportion of the bacterial species, favoring *S. mutans*, thus producing an increase in biomass due to augmented exopolysaccharide production, a result that has been replicated within this study (Koo et al., 2010). A direct correlation between sugar consumption and higher caries experience by DMFT index has recently been confirmed (Bernabé et al., 2016). Utilization of sucrose by *S. mutans* is the key cariogenic trait leading to the development of dental caries. The present study confirmed that as a sugar source sucrose was more cariogenic than galactose. *S. mutans* metabolizes sucrose into glucan, which is a component of the extracellular matrix. *S. mutans* is endowed with efficient metabolic mechanisms to better utilize free sugars over fermentable substrates, such as starch, thus promoting the formation of cariogenic biofilms.

Surprisingly, *S. mutans* strains within a polymicrobial biofilm environmental state do not conform to a specific pattern of growth. Significant differences were observed, and were not only dependent on the sugars, but also on specific isolates. Liu S.S. et al. (2017) revealed *S. mutans* isolates varied in their ability to compete and become dominant in the biofilm after the addition of sucrose (Liu Y. et al., 2017). Interactions between microorganisms colonizing the oral cavity are consecutive factors affecting the development of biofilm, with interactions occurring between microorganisms resulting in both the acceleration and inhibition of this process (Wen et al., 2010; Redanz et al., 2011). The compositional analysis described herein showed that *S. mutans* was the dominant species within the biofilm containing the caries isolate, while more *L. casei* were present in the biofilm containing the caries-free isolate. This could merely be coincidence, so we do not suggest that these outcomes from this model represent a predictor of carious potential. Instead, we present a model that enables us to investigate compositional and functional changes within complex biofilms, while also assessing functional changes driven by transcriptional changes. *S. mutans* may interact with *Lactobacillus* to accelerate or inhibit the process of biofilm formation depending on the isolate strains. *L. salivarius* strains with inhibitory activities on the growth and expression of *S. mutans* virulence genes have been shown to reduce its biofilm formation in many studies similar to data shown within (Wu et al., 2015; Krzysciak et al., 2017). *Lactobacillus* spp. inhibit the proliferation of the pathogen, and thus retard biofilm formation and development through LuxS and GtfB (Samot et al., 2011; Söderling et al., 2011; Ahmed et al., 2014). Conversely, it has been shown that biofilm formation by *S. mutans* significantly decreased when grown with *S. sanguinis*, but slightly increased when co-cultivated with *L. casei*, thus further highlighting the diversity and variability in isolates (Takahashi and Nyvad, 2011).

Real-time PCR was used to analyze the expression of genes that have critical roles in bacterial adherence and biofilm accumulation by *S. mutans* (Banas, 2004). Results show that the expression of these genes increased in polymicrobial biofilms containing caries isolates. It was identified that not only genes that are associated with sucrose metabolism (gtfB, ftf) were increased within the biofilm, but genes associated in non-sucrose metabolism (srtA, spaP, gbpB) were also enhanced. Elucidating that these are also involved in the utilization of sucrose and enhanced the cariogenic properties of the biofilm. Klein et al. (2012) showed that *S. mutans* adapts to a multi-species environment by changing the expression of genes associated with glucan synthesis, remodeling, and glucan-binding (Klein et al., 2012). In this way, *S. mutans* outcompetes other bacteria by optimizing its metabolism to a sucrose environment, thus increasing its competitiveness and thereby virulence. Collectively, this illustrates that cariogenic biofilms containing *S. mutans* are complex, and dependent upon the specific strains present thus driving different cariogenic phenotypes.

Biofilms are a multi-species complex attached on the tooth surface, with a myriad of consecutive factors contributing to biofilm development, which can result in both the acceleration and inhibition of this process (Simon-Soro et al., 2014). The study presented herein aims to provide a dynamic approach to studying the changes in biofilm under dietary pressure. This provides evidence that *S. mutans* in mixed-species communities interacts with other microorganisms depending upon the composition and specific phenotypes, thus driving dominant ecotypes. This is particularly important given the heterogeneity that exists within this species (Palmer et al., 2013; Yu et al., 2015). The virulence of *S. mutans* depends not only on the environmental conditions of the oral cavity, but also on the composition of the microbiome, and indeed the mycobiome (Ghannoum et al., 2010; Peters et al., 2017). Therefore, the model developed and presented offers useful and advantageous methodologies to test different experimental hypotheses related to caries research.

## Author Contributions

YZ, EM, and TS participated in the study design, carried out the experimental studies on biofilms, and performed statistical analysis; they were responsible for the manuscript. DL participated in study design, assisted with statistical support, and helped draft the manuscript. RR carried out the molecular studies on biofilms and helped draft the manuscript. JB and HL contributed to study design and supervised manuscript writing. GR conceived the study, participated in study design, data analysis, and was responsible for writing and submission of the final manuscript. All authors read and approved the manuscript.

## Conflict of Interest Statement

The authors declare that the research was conducted in the absence of any commercial or financial relationships that could be construed as a potential conflict of interest.

## Supplementary Material

FIGURE S1 *Streptococcus mutans* mono-species biofilm formation from clinical isolates is phenotypically variable. Standardized *S. mutans* isolates (1 × 10^7^ cells/mL) in BHI media with 0.25% sucrose were grown in flat-bottomed 96 well microtiter plates for 24 h at 37°C in 5% CO_2_. Mature biofilms were carefully washed with PBS, allowed to air dry and biomass quantified by staining with 0.05% w/v crystal violet solution. The biofilms were washed and de-stained with 100% ethanol. Biomass was quantified spectrophotometrically by reading absorbance at 570 nm in a microtiter plate reader (FluoStar Omega, BMG Labtech). Four replicates were used for each isolate and was carried out on triplicate separate occasions, with the mean of each represented.

FIGURE S2 Standardized *S. mutans* (ten isolates from caries and caries free patients) at 1 × 10^7^ cells/mL in BHI media containing different concentrations of sucrose, mannose, fructose, glucose, galactose (0.125, 0.25, 0.5, 1.0, 2.0, 5.0% w/v) for 24 h at 37°C in 5% CO_2_. Biomass crystal violet staining was quantified spectrophotometrically at 570 nm and supernatants collected and pH values quantified. Four replicates were used for each isolate (*n* = 3), with the mean of each represented. Data represents the mean.

FIGURE S3 Polymicrobial caries biofilm model containing standardized *L. casei*, *V. dispar*, *F. nucleatum*, *A. naeslundii*, and *S. mutans* isolates derived from either caries and caries-free patients grown at was grown in artificial saliva (AS) + galactose at 37°C in 5% CO_2_ for 5 days. Biofilms were assessed for total and viable composition of each bacterium using viability qPCR.

FIGURE S4 Polymicrobial caries biofilm model containing standardized *L. casei*, *V. dispar*, *F. nucleatum*, *A. naeslundii*, and *S. mutans* isolates derived from either caries and caries-free patients grown at was grown in artificial saliva (AS) + galactose at 37°C in 5% CO_2_ for 5 days. Total bacterial RNA was extracted and transcriptional analysis performed on *srtA*, *spaA*, *luxS*, *ftf*, *spaP* and *gpbB*. Gene expression was normalized to the housekeeping gene 16S rRNA according to 2^-ΔΔC_T_^ method and a heat map generated using the heatmap.2 function from the g-plots package.
