# Supplementary figures and images for: Evaluating Streptococcus mutans Strain Dependent Characteristics in a Polymicrobial Biofilm Community
Source: Front Microbiol. 2018 Jul 23;9:1498. doi: 10.3389/fmicb.2018.01498 (PMC6064717; doi:10.3389/fmicb.2018.01498)

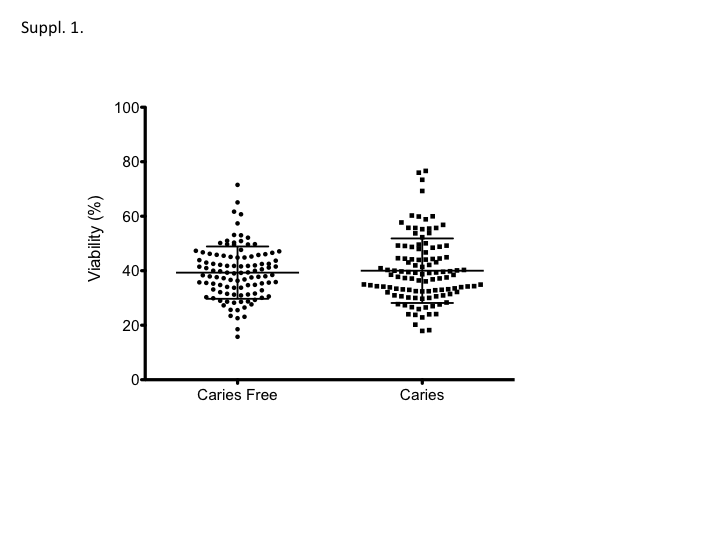

Supplement: FIGURE S1 [file Image_1.TIFF]

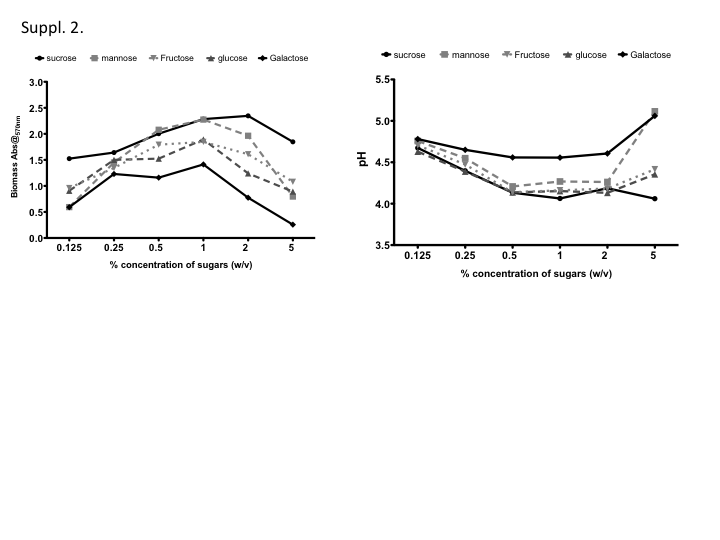

Supplement: FIGURE S2 [file Image_2.TIFF]

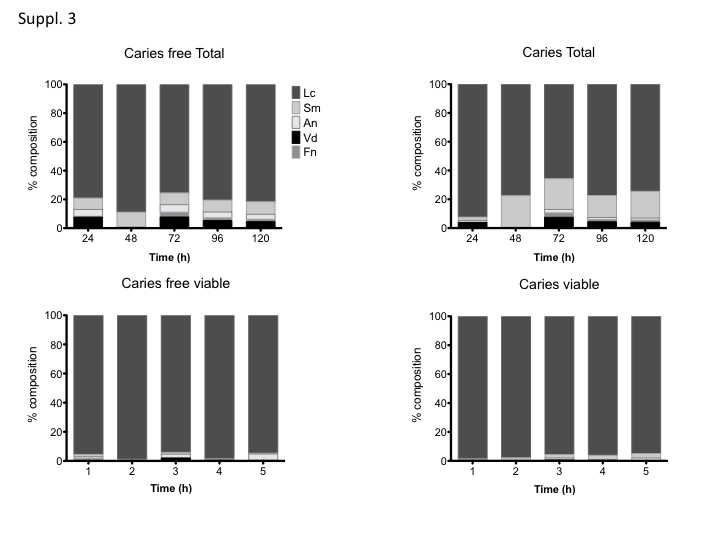

Supplement: FIGURE S3 [file Image_3.TIFF]

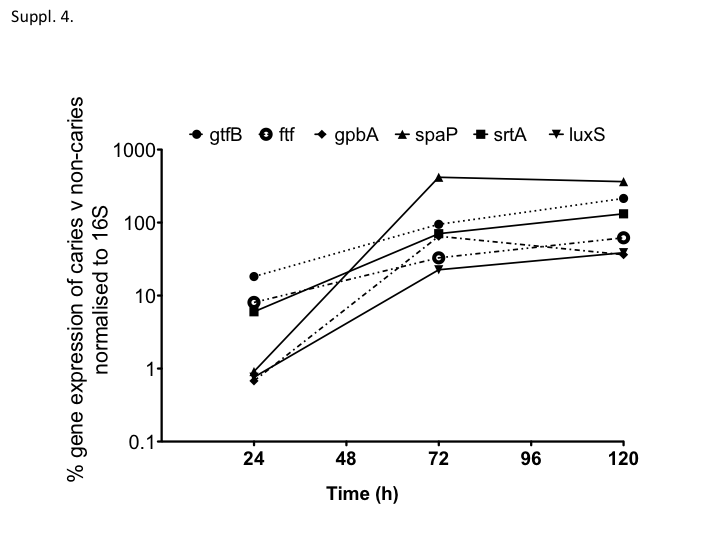

Supplement: FIGURE S4 [file Image_4.TIFF]
